# Eliminating Nonvalue-Added Tasks in Health Care: Artificial Intelligence in the Orthopedic Office

**DOI:** 10.1016/j.jhsg.2026.100992

**Published:** 2026-03-31

**Authors:** Thomas Doss, Jomar Aryee, Diana Vitkovska, Aryan Borole, Connor J. O’Leary, Marissa Viqueira, Daniel Okoh, Brian Katt

**Affiliations:** Department of Orthopaedic Surgery, Rutgers Robert Wood Johnson Medical School, New Brunswick, NJ

**Keywords:** Artificial intelligence, ChatGPT, Documentation, Note generation, Orthopedics

## Abstract

**Purpose:**

This study examines the efficiency and proof-of-concept use of a generative artificial intelligence (AI) documentation prototype, evaluating whether it can produce usable notes and estimating its potential time advantage compared with physician-produced documentation in an orthopedic practice.

**Methods:**

A comparative study analyzed AI-generated documentation versus physician-generated documentation of 68 new patient encounters with a board-certified hand surgeon, including surgical and nonsurgical patients presenting for evaluation of upper-extremity conditions. AI-generated documentation was created using unedited audiorecordings of encounters containing no identifiable patient information that were processed by standardized ChatGPT prompts. A senior resident (PGY-5) categorized all visits as minimal, low, or moderate complexity using the Centers for Medicare & Medicaid Services guidelines. Variables measured were documentation time (AI and physician), and physician time to edit AI documentation, visit complexity, visit time of day, patient demographics, and a physician-produced note quality assessment using a 5-point Likert scale.

**Results:**

Editing AI-generated documentation required a median of 55.1 seconds, compared with 81.2 seconds for physician-generated documentation. AI-generated notes were generally high quality, with 91% rated 4 or 5 on a 5-point Likert scale. Physician documentation time increased with greater visit complexity, whereas AI-documentation time showed minimal variation across complexity levels; complexity-stratified findings should be interpreted as exploratory because of sample size. Critical errors resulting in incomprehensible or erroneous content were identified in six (8.8%) AI-generated documents.

**Conclusions:**

This proof-of-concept study demonstrates that AI can produce clinically usable notes across varying encounter complexities and that physician editing of AI-generated documentation is faster than independent documentation. Although human oversight remains essential, AI-assisted documentation has substantial potential to improve workflow efficiency.

**Level of evidence:**

IV

The growing ubiquity of artificial intelligence (AI) across professional arenas contrasts sharply with the cautious optimism and relatively slow adoption seen in the field of medicine. From the radiology reading room to the orthopedic clinic, AI has the potential to become a viable partner to physicians in ways yet to be fully explored.^1^ One promising application of this technology is the use of generative AI-powered tools such as ChatGPT that can decrease the burden of nonvalue-added tasks in health care. In health-care operations and workflow efficiency models, “nonvalue-added tasks” refer to activities that are necessary for administrative or communication purposes but do not directly contribute to improving patient health outcomes. Although clinical documentation is essential for communication, billing, and longitudinal patient care, the physical act of entering information into the medical record does not itself improve patient outcomes and therefore is classified as a nonvalue-added task. In recent years, most health-care professionals have identified it as unduly cumbersome, time-consuming, and a leading contributor to the increased rates of burnout and exhaustion.^2^, ^3^, ^4^, ^5^ Many have turned to dictation services, personal scribes, and other modes of data entry to minimize the impact of documentation and related administrative tasks on time spent delivering patient care. The efficiency, benefits, and pitfalls of the various modes of data entry into electronic health records have been studied.^6^ The documentation of patient encounters into the medical record is one nonvalue-added task where a generative AI tool could have an immediate impact. A recent study has shown that AI-powered documentation tools can meaningfully reduce documentation burden and electronic health record (EHR)-related frustration among clinicians.^7^

The purpose of this proof-of-concept study is to assess whether a generative AI prototype can produce clinical documentation of comparable quality to physician-generated notes and to estimate the potential speed advantage of AI-generated documentation. This work aims to provide insight into the value of AI in the field of medicine and allow readers to grasp the yet unrealized potential of this technology in their practice. A head-to-head comparison of physician-generated and AI-generated clinical documentation for new patient encounters seen by a single, board-certified, fellowship-trained hand surgeon was performed to produce the data for our analyses. The authors of this study primarily hypothesize that for new patient encounters, it will be significantly faster for a board-certified hand surgeon to read and edit documentation of the history, physical examination, and assessment/plan produced by a generative AI model than to document the encounter themselves. The authors secondarily hypothesize that (1) the level of complexity of a patient’s presenting problem will have no effect on the difference between the time taken to read and edit documentation produced by a generative AI model and the time a physician takes to document an encounter and (2) the length of a patient’s encounter will have no effect on the difference between the time taken to read and edit documentation produced by a generative AI model and the time a physician takes to document an encounter.

## Methods

### Study design

This head-to-head comparative, proof-of-concept study examined physician-generated versus AI-generated documentation for new patient encounters conducted by a single, board-certified hand surgeon. Encounters included both surgical and nonsurgical patients presenting with hand and upper-extremity conditions. The study aimed to evaluate the time efficiency of AI-generated documentation compared with traditional physician-generated documentation, as well as the quality of AI-generated documentation. The authors state that this research was conducted in accordance with the most recent iteration of the Helsinki Declaration. Informed consent was obtained from all patients prior to participation in and for publication of this study.

### AI-documentation generator

An AI-based documentation generator was developed using ChatGPT-3.5 (2022) in late 2023–early 2024, when this model represented the most stable and accessible option for API-based integration. A workflow that converted audiorecordings of clinical encounters into documentation was created using Pipedream (2019), a cloud-based integration platform that orchestrates API calls through a graphical workflow, Google Drive (2012), and ChatGPT-3.5 “Whisper” (2022).

For each encounter, an audiorecording was uploaded to a designated Google Drive folder, triggering a Pipedream workflow that retrieved the file, transcribed the audio using Whisper, and passed the transcript to the ChatGPT-3.5 API to generate clinical documentation. The output was returned as a text document for physician review and editing. No custom model training or fine-tuning was performed; all steps were configured using standard connectors with minimal inline code.

Documentation prompts for ChatGPT-3.5 were developed using publicly accessible recordings of standardized patient musculoskeletal cases. A senior orthopedic resident created reference Subjective, Objective, Assessment, and Plan (SOAP) notes that served as quality benchmarks. Prompts were refined until the AI-generated documentation demonstrated comparable structure completeness to the resident-generated notes. Two fixed prompts were finalized: (1) a transcription prompt to convert audio to text and (2) a documentation prompt directing the model to generate a narrative history followed by a structured “SOAP” note including pertinent subjective and objective findings, modifying factors, and a summary with an impression and plan. These prompts were applied uniformly to all 68 encounters. Full prompt text and a link to the standardized patient case are provided in the Appendix S1, available online on the Journal’s website at https://www.jhsgo.org.

AI-generated notes followed standard “SOAP” structure and included a descriptive title, history, physical examination findings, assessment, and plan. They concluded with a summary paragraph reiterating the patient’s age, gender, and key symptoms, along with a differential diagnosis and a proposed treatment plan. Physical examination findings were captured as the provider purposefully said them aloud during the patient encounter, which differs from the surgeon’s usual documentation practice.

### Data collection

Institutional Review Board approval was obtained (IRB No. Pro2024001540). A total of 68 new patient encounters were recorded between August 22, 2024, and October 3, 2024. Verbal consent was obtained from all patients. Encounters were audiorecorded using the Voice Memos application on an Apple iPhone 15. A research staff member was present solely to record the encounter and did not participate in or influence the clinical interaction.

No protected health information (PHI), as defined by the Health Insurance Portability and Accountability Act (45 CFR §164.514), was recorded or stored.^8^ The physician deliberately avoided verbalizing PHI identifiers such as patient names, dates of birth, medical record numbers, prior surgical dates, or other temporal references that could link data to a specific patient. Each recording was assigned a unique study identifier, and because no PHI was captured, Health Insurance Portability and Accountability Act-compliant file storage was not required.

*Encounter length* was defined as the duration of the audiorecording and included history taking, physical examination, and discussion of assessment and plan, but excluded procedural care (eg, injections, casting). After each visit, the data collector supplemented the recording with patient age and sex and uploaded the recording to a secure institutional cloud drive for AI processing. The time required for uploading and adding identifiers was not recorded. *AI-documentation time* was defined as the time required for the AI system to generate a transcript and “SOAP” note from the audiorecording.

The physician edited AI-generated notes using Microsoft Word’s “dictate” feature (1983). *Physician time to edit AI-documentation* was defined as the time required to revise the AI-generated note to match the same standard of completeness and accuracy as the surgeon’s routinely produced clinical documentation. Physician-generated notes were completed immediately after each encounter, consistent with usual practice. Editing of AI-generated notes occurred either at the midpoint of the clinic session or at the end of the day.

AI-generated note quality was assessed by the physician using a subjective, nonvalidated 5-point Likert scale evaluating completeness, consistency, and readability as follows:

• 5: Fully complete, requiring no additions to the history, diagnosis, or plan; consistent with the patient-physician conversation; and logically ordered.
• 4: Near-complete, missing no more than two facts; logical order with minor syntax or verbiage edits needed in no more than two sentences.
• 3: Near-complete but missing more than two facts; includes at least one inconsistency with the conversation.
• 2: Incomplete, missing more than two facts; contains multiple inconsistencies with the conversation; difficult to understand, requiring edits in more than two sentences.
• 1: Requires complete rewriting.

After all the patient documentation was completed (68 encounters), a fifth-year resident independently reviewed physician-generated documentation and assigned *visit complexity* to each encounter using the Centers for Medicare & Medicaid Service medical decision-making guidelines and a structured rubric.^9^ This resident routinely applies the Centers for Medicare & Medicaid Service complexity framework in clinical practice as part of standard orthopedic documentation training. The time required for the complexity assignment was not recorded.

### Statistical analysis

Dependent variables included *physician documentation time*, *AI-documentation time*, *physician time to edit AI documentation*, and the difference between *physician documentation time* and *physician time to edit AI documentation*. *Visit complexity*, *encounter length, visit time of day (am or pm), and patient age and sex* represented the independent variables.

### Data analysis

The data were analyzed to evaluate for differences between *physician documentation time* and *physician time to edit AI documentation*, stratifying these data based on independent variables including *visit complexity*, visit time of day, and patient age and gender. A 2-sample *T* test was used to determine the difference between physician documentation time and physician time to edit AI documentation stratified by time of visit.

The Kruskal-Wallis test was conducted to detect differences between documentation times (physician and AI) among all minimal, low, and moderate complexity groups together. If the Kruskal-Wallis result was significant with *P* < .05, the pairwise Wilcoxon rank-sum test was performed to determine differences in any two complexity levels.

Statistical analyses were conducted using R Studio (version 4.1.3), with a *P* value of less than 0.05 considered statistically significant.

## Results

### Demographics

There were 68 patients with an average age of 54.7 ± 21.8 years. Thirty-six patients (53%) were women, and 35 visits (51%) occurred in the afternoon (Table 1).

**Table 1:** Patient Characteristics

Variable	No. of Patients (n)	No. of Patients (%)
Sample size (n)	68	100%
AM versus PM visit		
AM	33	49%
PM	35	51%
Sex (n)		
M	32	47%
F	36	53%
Age (n)		
<30 y	11	16%
31–60 y	23	34%
61–90 y	32	47%
>90 y	2	3%
Visit complexity (n)		
Minimal/straightforward	9	13%
Low	41	60%
Moderate	18	26%

### Physician documentation time versus physician time to edit AI documentation

There was a statistically significant difference in the time between the physician-generated documentation time and the physician-edit time of AI documentation (81.2 ± 17.4 vs 55.1 ± 28.8 s; *P* < .001). This difference remained significant regardless of the time of day (Table 2).

**Table 2:** Clinical Documentation Production Time

Variable	Physician documentation time (s)	Physician time to edit AI documentation (s)	*P* value
Overall (mean ± SD)	81.2 ± 17.4	55.1 ± 28.8	<.001
AM versus PM visit			
AM	85.1 ± 19.2	66.1 ± 29.4	.002
PM	77.6 ± 15.0	44.8 ± 24.4	<.001

### Patient/visit complexity

Of the 68 patients, 9 patients were graded as minimal/straightforward complexity, 41 low complexity, and 18 moderate complexity visits. Median physician documentation time, AI-documentation time, and median visit times were stratified by visit complexity (Table 3). As complexity increased from straightforward/minimal to low and moderate, median physician documentation time increased from 64.9 to 79.0 seconds and 88.9 seconds, respectively (*P* = .048). In contrast, AI-documentation times did not show a statistically significant difference among straightforward (40.5 seconds), low (43.5 seconds), and moderate (47.5 seconds) complexities (*P* = .14). Additionally, median visit times increased with complexity, from 213 seconds for straightforward cases to 265 seconds and 337 seconds for low and moderate complexities (*P* = .009). Although visit duration did not differ significantly between straightforward and low complexity visits (*P* = .207), moderate complexity visits were longer than both low complexity (*P* = .03) and straightforward complexity visits (*P* = .01) (Table 3). Subgroup analyses are exploratory because of limited sample sizes, particularly in the minimal-complexity subgroup (n = 9).

**Table 3:** Effects of Patient Complexity on Documentation and Visit Times

Complexity	Median physician documentation time	Median AI-documentation time	Median visit time
Straightforward/ minimal	64.9 s	40.5 s	213 s
Low	79.0 s	43.5 s	265 s
Moderate	88.9 s	47.5 s	337 s
	*P* = .048	*P* = .14	*P* = .009

### AI note time and quality

Across all encounters, AI-generated notes received a mean quality rating of 4.3 out of 5 (SD ± 0.8; range 1–5) using the subjective 5-point Likert scale described previously (Tables 2, 4). The average time for AI note generation was 48.4 ± 14.2 s. Six of the 68 AI-generated notes (8.8%) received a score of ≤3 because of critical documentation inaccuracies. Errors were categorized as either incorrect representation of non-PHI encounter details (eg, age, gender, chief complaint) or hallucinated content. In five of the six low-scoring notes, the AI misattributed unrelated background words or fragmented conversation to key components of the SOAP note. In one note, the model produced a repeatedly inserted 3-word phrase that had never been spoken during the encounter, representing a clear hallucination.

**Table 4:** Physician-Assigned Quality Ratings of AI-Generated Documentation Using a 5-Point Likert Scale

Score	No. of visits (n)	No. of visits (%)
1	1	1
2	1	1
3	4	6
4	34	50
5	28	41

## Discussion

This proof-of-concept study demonstrates that generative AI can consistently document new clinical encounters of varying complexity and reduce the time physicians spend on documentation of new patient encounters.

Physician time to edit AI-generated documentation (55.1 ± 28.8 seconds) was 33% shorter than the time required to produce documentation from scratch (81.2 ± 17.4 seconds). Extrapolating to a clinic volume of 40 new encounters, this would correspond to approximately 17 minutes saved per clinic day. These findings counter the concern that overseeing AI output might be more time-consuming than writing new notes. Additionally, this study suggests that a generative AI can produce consistent documentation across varying patient complexity levels. When stratified by visit complexity, physician documentation time increased with increasing complexity, whereas AI-documentation time did not significantly differ. Although subgroup analyses are exploratory because of small sample sizes, especially in minimal-complexity visits, the pattern suggests that generative AI tools may offer consistent documentation speed regardless of encounter complexity.

The use of AI in medicine is expanding rapidly.^1^ In a similar study, ChatGPT-generated orthopedic-discharge notes achieved comparable quality to physician notes while being produced 10 times faster.^10^ Another study used a web-based electronic data capture system that merged structured clinical data to generate template-based EHR notes, reducing documentation time from 22.4 to 7.1 minutes per patient.^11^ AI-generated operative notes demonstrated accuracy comparable with surgeon-written documentation, although concerns regarding completeness remain.^12^

Reducing clinical documentation time is critical in addressing physician burnout, which is associated with depression, substance use, reduced quality of care, and attrition.^13^, ^14^, ^15^, ^16^ Interventions targeting burnout have been shown to improve well-being, work engagement, depression, and anxiety.^14^^,^^17^ Because burnout requires a multimodal approach,^18^^,^^19^ reducing documentation burden with AI may be an important early step, enabling physicians to focus more on patient care and potentially improving patient-physician relationships and outcomes. Recent clinical trial data support this concept, demonstrating that AI-powered documentation tools were associated with reduced time spent on documentation, decreased after-hours EHR use, and lower clinician frustration.^7^

In this study, 91% of AI-generated notes received a quality rating of 4 or 5 out of 5, with an overall mean score of 4.3 (SD ± 0.8). These findings demonstrate that even a low-cost, non-electronic medical record (EMR)-integrated prototype built on an older model could produce documentation that required minimal physician editing and remained comparable in quality to physician-generated notes. However, important ethical considerations remain. A key concern is the potential for AI-generated errors or “hallucinations,” in which incorrect or fabricated information is inserted into the medical record when the model attempts to fill gaps created by incomplete or low-quality input.^5^^,^^20^ In this study, 6 AI-generated notes (8.8%) contained critical inaccuracies such as misattributed background conversation or incorrect non-PHI demographic details, typically in encounters with low speech volume or environmental noise. Even with these errors, physician editing time remained significantly shorter than independent documentation. These findings are not unusual; a recent systematic review of AI-powered documentation systems found that although AI improves documentation efficiency, the presence of hallucinations remain key patient safety limitation requiring continued physician oversight.^5^ Maintaining stringent data security protocols is crucial for protecting patient confidentiality and trust.^21^^,^^22^

Importantly, this study did not account for the time required to move from recorded audio to an AI-generated note. It is not the goal of the authors to imply that this time was negligible, but instead to highlight that with automations such as direct integration of AI-documentation tools within EMR, the benefits of using such an integrated technology could be substantial. However, a recent study showed that AI integration may shift documentation tasks to after-hours EHR use when AI-generated drafts are not available for real-time review and signature, underscoring that AI may redistribute rather than eliminate documentation workload.^22^ Although EMR-embedded systems (eg, Abridge in Epic) now exist, such tools were not available in the authors’ practice environment during study design and data collection.

Because the AI system depends on verbalized examination findings, clinicians may need to adapt their encounter style to ensure all necessary information is spoken aloud. In this study, the surgeon verbalized examination findings for consistency, differing from routine practice. Although this may have subtly influenced the flow of the live encounter, it did not affect the documentation time variables measured in this study, which were limited to postencounter note creation and editing.

A potential Hawthorne effect should be considered, as in this study, physician documentation and editing times were recorded without blinding. Awareness of being observed could theoretically influence pace. However, both physician-generated and AI-documentation editing were timed under identical conditions. Thus, any such influence would apply uniformly across all notes and would not be expected to bias the comparison between the two methods. Future blinded, multievaluator studies would strengthen these findings.

### Limitations

This study involved a single physician and a modest sample size, limiting generalizability. Other providers with different documentation styles or comfort with AI may experience different degrees of benefit. Complexity-stratified analyses should be considered exploratory because of small subgroup sample sizes. Additionally, although physician documentation times were relatively fast in this study, AI assistance still yielded time savings, reinforcing the potential value of this approach. Another limitation is the narrow focus of this study, as it did not aim to evaluate more complex documentation requirements, such as integrating laboratory data, imaging interpretation, or reconciling prior records, tasks that remain significant contributors to physician workload.

The quality of AI-generated notes was assessed using a subjective 5-point Likert scale by a single hand surgeon. This scale is not validated, and ratings may reflect individual preference rather than universally applicable standards. Future studies should use multiple independent reviewers and validated scoring tools to improve reproducibility. The AI-documentation prototype used in this study was developed before the release of newer models such as GPT-4o and GPT-5. At the time, ChatGPT-3.5 was the most stable and accessible option for API-based integration, with predictable formatting and reliable compatibility with the Whisper speech-to-text pipeline. Future studies should reassess performance with updated models.

## Conflict of Interest

No benefits in any form have been received or will be received related directly to this article.
