# Supplementary material for: Eliminating Nonvalue-Added Tasks in Health Care: Artificial Intelligence in the Orthopedic Office
Source: J Hand Surg Glob Online. 2026 Mar 31;8(3):100992. doi: 10.1016/j.jhsg.2026.100992 (PMC13068648; doi:10.1016/j.jhsg.2026.100992)
Supplement: Appendix S1 [file mmc1.docx]

Finalized Prompts used in the study:

1. This is an audio file of a patient encounter with an orthopedic surgeon. Create a transcript of this conversation.
2. Then return a paragraph using the transcription as the source of information of this encounter that creates a history beginning with a sentence with the age and gender of the patient and includes their chief complaint and reason for visit. Then generate a SOAP note appropriate for a medical record using the transcription of this encounter. The SOAP note should encompass all pertinent information based on the transcription and includes the quality of symptoms, time of onset of symptoms, frequency of symptoms, location of pain or symptoms, severity of symptoms and limitations they cause, presence or absence of any pertinent associated symptoms, and any symptom modifying activities, medications or factors. End the transcription with a small paragraph summary that once again begins with the age, gender, and pertinent symptoms (in that order) and includes a most likely diagnosis or a differential diagnosis and includes a corresponding plan that states the treatment of the current problems and additional steps for a definitive diagnosis.

Prompt iterations:

**Prompt 1**

Then return a paragraph using the transcription of this encounter that creates a history beginning with a sentence with the age and gender of the patient and includes their chief complaint and reason for visit and formats the transcription in a format which includes the quality of symptoms, time of onset of symptoms, frequency of symptoms, location of pain or symptoms, severity of symptoms and limitations they cause, presence or absence of any pertinent associated symptoms, and any symptom modifying activities, medications or factors. End the transcription with a one to two sentence assessment with the age, gender, and pertinent symptoms (in that order) and includes a most likely diagnosis or a differential diagnosis then create a small paragraph that includes the treatment of the current problems and additional steps for a definitive diagnosis.

**Output 1**

Elizabeth, a 58-year-old female, presented with a chief complaint of lower back pain that began five days ago following a minor fall. The pain was initially dull and achy but has since become more acute, radiating anteriorly to the top of her thigh, accompanied by tingling sensations down to her knee. The pain has increased in severity, prompting her visit. It is exacerbated by movement and has not significantly improved with ibuprofen. Elizabeth has a history of chronic low back pain due to previous injuries and osteoarthritis. She denies any recent changes in bowel or bladder function, or other systemic symptoms. Given her symptoms and history, a musculoskeletal origin is likely, with differential diagnoses including nerve impingement or exacerbation of previous lumbar disc herniation.

Treatment involves imaging studies, starting with an X-ray, to assess for structural causes. Elizabeth was advised to use a back brace and diclofenac cream for localized pain management, and to avoid strenuous activities. Further management will depend on imaging results, with potential escalation to MRI if needed.

**Prompt 2**

Then return a SOAP note appropriate for a medical record using the transcription of this encounter beginning with a sentence with the age and gender of the patient and includes their chief complaint and reason for visit. The SOAP note should encompass all pertinent information based on the transcription and includes the quality of symptoms, time of onset of symptoms, frequency of symptoms, location of pain or symptoms, severity of symptoms and limitations they cause, presence or absence of any pertinent associated symptoms, and any symptom modifying activities, medications or factors. End the transcription with a one to two sentence assessment that once again begins with the age, gender, and pertinent symptoms (in that order) and includes a most likely diagnosis or a differential diagnosis then create a corresponding plan that includes the treatment of the current problems and additional steps for a definitive diagnosis.

**Output 2**

**Subjective:**

- **Patient Information:** Elizabeth, a 57-year-old female.

- **Chief Complaint:** Lower back pain worsening over five days following a low fall.

- **History of Present Illness:** Pain started dull and achy, now radiates anteriorly with tingling sensations to the knee. No head injury or loss of consciousness during the fall. Pain exacerbated by movement, partially relieved by ibuprofen.

- **Past Medical History:** Chronic low back pain from a car accident and martial arts injury, osteoarthritis, high cholesterol treated with simvastatin.

- **Medications:** Ibuprofen, simvastatin, Flomax, Celebrex (as needed).

- **Social History:** Lives with wife and three children, works as a landscaper.

**Objective:**

- **Physical Examination:** Pain on palpation of lower back and sacrum, limited range of motion in back and hips, positive straight leg test on the left, normal neurological examination in lower extremities.

- **Vital Signs:** Not provided in the transcript.

**Assessment:**

- **Preliminary Diagnosis:** Acute exacerbation of chronic low back pain, possible nerve impingement or disc herniation.

- **Differential Diagnosis:** Sciatica, lumbar strain, underlying osteoarthritis exacerbation.

**Plan:**

- **Immediate Management:** Prescribe diclofenac cream for localized anti-inflammatory effect, advise rest and avoidance of strenuous activities, use of a back brace for support.

- **Diagnostic:** Order x-rays of the lumbar spine and hips, review previous imaging studies for changes.

- **Follow-Up:** Schedule a follow-up appointment post-imaging review, consider MRI if x-rays are inconclusive.

- **Patient Education:** Educate on proper back care and the importance of gradual resumption of activities.

**Prompt 3**

Then return a SOAP note appropriate for a medical record using the transcription of this encounter beginning with a standalone sentence with the age and gender of the patient and includes their chief complaint and reason for visit, in that order. The SOAP note should encompass all pertinent information based on the transcription and includes the quality of symptoms, time of onset of symptoms, frequency of symptoms, location of pain or symptoms, severity of symptoms and limitations they cause, presence or absence of any pertinent associated symptoms, and any symptom modifying activities, medications or factors. End the transcription with a small paragraph assessment that once again begins with the age, gender, and pertinent symptoms (in that order) and includes a most likely diagnosis or a differential diagnosis then create a corresponding plan that includes the treatment of the current problems and additional steps for a definitive diagnosis.

**Output 3**

**Patient Information:**

- **Age/Gender:** Not specified in the transcript

- **Chief Complaint:** Acute lower back pain following a fall

- **History of Present Illness:** The patient reports a low fall five days ago, resulting in progressive lower back pain with tingling sensations radiating to the thigh. The pain has been worsening despite rest and ibuprofen intake.

**SOAP Note:**

**Subjective:**

- The patient reports acute lower back pain starting five days ago after a minor fall. The pain has progressively worsened, now radiating with tingling sensations to the left thigh. The patient denies any head injury, loss of sensation beyond the knee, or changes in bowel or bladder function.

**Objective:**

- Physical examination reveals tenderness in the lower back with limited range of motion during flexion, hyperextension, and lateral movements. Sensation and reflexes are intact. No signs of acute distress or neurological deficits were noted.

**Assessment:**

- A middle-aged patient presents with acute lower back pain and radiating tingling sensations following a minor fall. Considering the patient's history of chronic back issues and the nature of the symptoms, differential diagnoses include nerve impingement or exacerbation of previous lumbar disc herniation.

**Plan:**

- **Imaging:** Order x-rays of the lumbar spine and possibly an MRI if x-rays are inconclusive.

- **Medication:** Prescribe diclofenac cream for localized anti-inflammatory treatment and continue ibuprofen as needed, monitoring for gastrointestinal side effects.

- **Supportive Care:** Recommend a back brace and advise on proper pillow positioning during sleep to prevent pain exacerbation.

- **Follow-up:** Schedule a follow-up appointment to review imaging results and adjust the treatment plan as necessary. Advise the patient to avoid strenuous activities and landscaping work until further evaluation.

In summary, the patient, a middle-aged individual experiencing worsening lower back pain with radiating symptoms following a fall, likely has nerve impingement or an exacerbation of a previous lumbar condition. Immediate imaging and supportive care are recommended, with a follow-up to adjust the treatment plan based on imaging results.

**Prompt 4**

Then return a paragraph using the transcription of this encounter that creates a history beginning with a sentence with the age and gender of the patient and includes their chief complaint and reason for visit. Then generate a SOAP note appropriate for a medical record using the transcription of this encounter. The SOAP note should encompass all pertinent information based on the transcription and includes the quality of symptoms, time of onset of symptoms, frequency of symptoms, location of pain or symptoms, severity of symptoms and limitations they cause, presence or absence of any pertinent associated symptoms, and any symptom modifying activities, medications or factors. End the transcription with a small paragraph assessment that once again begins with the age, gender, and pertinent symptoms (in that order) and includes a most likely diagnosis or a differential diagnosis then create a corresponding plan that includes the treatment of the current problems and additional steps for a definitive diagnosis.

**Output 4**

### Subjective:

A 50-year-old male landscaper presents with a chief complaint of lower back pain that started five days ago after a low fall at home. He reports the pain as dull and achy initially, now radiating anteriorly with tingling sensations to the left buttock and thigh, not extending beyond the knee. The pain has worsened despite taking 600 mg of ibuprofen twice daily since day four. He denies any head injury, loss of sensation beyond the knees, or changes in bowel or bladder function. Past medical history includes chronic low back pain from a previous car accident, mild herniation, martial arts injury, and diagnosed osteoarthritis. He is currently on simvastatin for high cholesterol and Flomax and Celebrex as needed.

### Objective:

Physical examination shows limited flexion and hyperextension of the back with pain, as well as pain on lateral movement to the left side. Sensation and reflexes are intact. No crepitus or signs of acute fracture found on palpation.

### Assessment:

50-year-old male with worsening lower back pain following a fall, likely exacerbated by underlying chronic back issues and mild herniation. Differential diagnosis includes acute soft tissue injury, nerve impingement, or exacerbation of pre-existing back conditions.

### Plan:

1. Order x-rays of the lumbar spine and possibly an MRI if no improvement or further symptoms develop.

2. Prescribe diclofenac cream for localized anti-inflammatory treatment.

3. Advise rest, avoiding strenuous activities, and using a back brace to limit movement and prevent further injury.

4. Follow-up appointment after imaging results are available to adjust treatment plan as necessary.

This plan aims to manage pain, prevent further injury, and diagnose any potentially serious underlying conditions contributing to the patient's symptoms.

**Prompt 5 (Final prompt used in the study)**

Then return a paragraph using the transcription as the source of information of this encounter that creates a history beginning with a sentence with the age and gender of the patient and includes their chief complaint and reason for visit. Then generate a SOAP note appropriate for a medical record using the transcription of this encounter. The SOAP note should encompass all pertinent information based on the transcription and includes the quality of symptoms, time of onset of symptoms, frequency of symptoms, location of pain or symptoms, severity of symptoms and limitations they cause, presence or absence of any pertinent associated symptoms, and any symptom modifying activities, medications or factors. End the transcription with a small paragraph summary that once again begins with the age, gender, and pertinent symptoms (in that order) and includes a most likely diagnosis or a differential diagnosis and includes a corresponding plan that states the treatment of the current problems and additional steps for a definitive diagnosis.

**Output 5**

A 57-year-old male landscaper presents with a chief complaint of lower back pain that started five days ago after a low fall at home. He reports that the pain has increased in severity, now radiating to the front of his thigh and down to his knee, accompanied by tingling but no loss of sensation or motor function. His medical history is significant for chronic low back pain secondary to a previous car accident and martial arts injury, osteoarthritis, and high cholesterol treated with simvastatin.

## SOAP Note

**Subjective:**

- The patient reports acute lower back pain starting five days ago after a fall.

- Pain has worsened, now radiates anteriorly with tingling.

- Previous history of chronic back issues and osteoarthritis.

- Currently taking simvastatin, Flomax, and Celebrex as needed.

**Objective:**

- Physical examination shows tenderness in the lower back with limited range of motion.

- Neurological examination is normal; reflexes are intact.

- No signs of acute distress or systemic illness.

**Assessment:**

- Acute exacerbation of chronic lower back pain, likely musculoskeletal in origin.

- Differential diagnoses include nerve impingement or minor vertebral injury.

**Plan:**

- Order x-rays of the lumbar spine to rule out fractures or significant structural changes.

- Prescribe diclofenac cream for localized anti-inflammatory treatment.

- Advise continued rest, avoiding strenuous activities, and using ice packs.

- Follow-up appointment after imaging results are available.

A 57-year-old male with a history of chronic back issues now presents with worsening lower back pain following a minor fall, likely indicating an acute exacerbation of a pre-existing condition. Initial management includes imaging studies, topical medication, and conservative measures with planned follow-up.

Standardized patient case link:

<https://www.youtube.com/watch?app=desktop&v=wKvaAMr3GKs&feature=youtu.be>
